# Some Problems in Proving the Existence of the Universal Common Ancestor of Life on Earth

**DOI:** 10.1100/2012/479824

**Published:** 2012-04-30

**Authors:** Takahiro Yonezawa, Masami Hasegawa

**Affiliations:** ^1^School of Life Sciences, Fudan University, Shanghai 200433, China; ^2^Department of Statistical Modeling, Institute of Statistical Mathematics, Tokyo 190-8562, Japan

## Abstract

Although overwhelming circumstantial evidence supports the existence of the universal common ancestor of all extant life on Earth, it is still an open question whether the universal common ancestor existed or not. Theobald (Nature 465, 219–222 (2010)) recently challenged this problem with a formal statistical test applied to aligned sequences of conservative proteins sampled from all domains of life and concluded that the universal common ancestor hypothesis holds. However, we point out that there is a fundamental flaw in Theobald's method which used aligned sequences. We show that the alignment gives a strong bias for the common ancestor hypothesis, and we provide an example that Theobald's method supports a common ancestor hypothesis for two apparently unrelated families of protein-encoding sequences (*cytb* and *nd2* of mitochondria). This arouses suspicion about the effectiveness of the “formal” test.

## 1. Introduction

Data generated by genomic sequencing projects from a wide variety of species now allow for the assembly of combined protein sequence data sets to reconstruct the universal tree of life (e.g., [1]). On the other hand, it is still an open question whether the universal common ancestor (UCA) of all extant life on Earth existed or not. Although molecular phylogenetic methods automatically construct a tree when a sequence data set is provided, the inferred tree does not necessarily guarantee the existence of UCA, because its existence is assumed implicitly from the beginning usually in molecular phylogenetics.

 The theory of UCA has enjoyed a compelling list of circumstantial evidence as given by Theobald [2]. However, there had been no attempt to test the UCA hypothesis among three domains (or superkingdoms) of life, that is, eubacteria (Bacteria), archaebacteria (Archaea), and eukaryotes (Eukarya), by using molecular sequences until Theobald [2] challenged this problem with a formal statistical test. By using the sequence data sets compiled by Brown et al. [1] and by using the model selection criterion AIC [3], he showed that the UCA hypothesis is much superior to any independent origin hypothesis, and he concluded that the UCA theory holds. While the UCA hypothesis postulates that eubacteria, archaebacteria, and eukaryotes descended from a single common ancestor called UCA, the independent origin hypotheses include scenarios such as eubacteria having a different origin from that of archaebacteria/eukaryotes or the three domains have different origins from each other. His attempt is the first step towards the goal of establishing the UCA theory with a solid statistical ground. However, his methodology contains some problems for establishing the UCA theory as discussed by us [4], and, in this communication, we will give further details of our arguments.

The most serious problem of Theobald's analysis is that he used aligned sequences compiled by Brown et al. [1], who were interested in resolving the phylogenetic relationships among archaebacteria, eubacteria, and eukaryotes, including whether each domain of life constitutes a monophyletic clade. So they a priory assumed the existence of UCA. Indeed, alignment is a procedure based on an assumption that the sequences have diverged from a common ancestral sequence. Brown et al. wrote “Individual protein families were first computer aligned and then we manually refined the alignments. We removed poorly conserved regions in individual protein alignments.” This procedure clearly assumes the existence of UCA, and this was not a problem for Brown et al., because what they were interested in was the phylogenetic relationship among all species on Earth, and the existence of UCA was supported by circumstantial evidence [2]. However, in proving the existence of UCA, the alignment procedure should not be used, because it gives a strong bias for the UCA hypothesis.

 In a previous communication [4], we provided an example from two apparently unrelated families of nucleic acid coding sequences (*cytb* and *nd2* of mitochondria) for which AIC chooses a common origin hypothesis. Since alignment gives a bias for common ancestry, we did not make an alignment between *cytb* and *nd2*, but still the common origin of *cytb* and *nd2 *was preferred to the independent origins of these two genes. Probably no one will believe that this result should be regarded as evidence of the ultimate common ancestry of *cytb* and *nd2*. Rather this raises a question mark as to the effectiveness of Theobald's test.

Theobald [5] criticized our analysis by pointing out that our nucleotide substitution model of GTR+Γ is too naïve. We used the same reading frame of the two genes, but, according to Theobald, the constraints of the genetic code are expected to induce correlations between these sequences that are not due to common ancestry. This is a good point, and in this work we will use the amino acid substitution model as well to account of this correlation. We used only the GTR+Γ model of nucleotide substitution in [4] in order to show the most impressive case without alignment, but actually the preference of the common origin model over the independent origin model depends on the assumed substitution model. Therefore, by using several alternative substitution models of nucleotides as well as amino acids, we will study whether default settings of the alignment program, with which the data set of Theobald was made, reject the common origin hypothesis of the two apparently unrelated genes.

## 2. Materials and Methods

**Figure 1:**
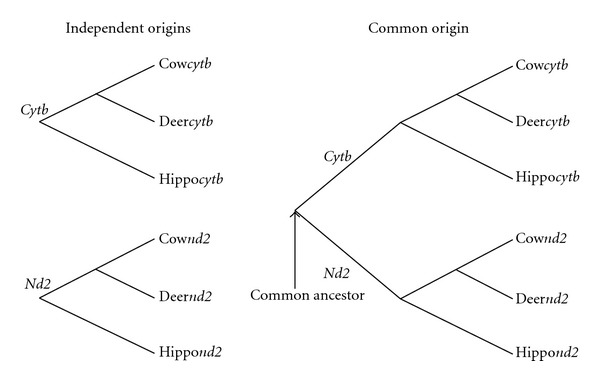
Independent origins hypothesis versus common origin hypotheses of *cytb* and *nd2*. No branch exists connecting the two genes in the independent origins hypothesis, while the common ancestor of the two genes exists in the common origin hypothesis.

The same sequence data set as used in [4] was provided for the analyses. The 5′-terminal 1,038 bp (excluding the initiation codon) of mitochondrial genes of *cytb* and *nd2* from cow (EU177848), deer (AB210267) and hippopotamus (NC_000889) was analyzed by the maximum likelihood method implemented in PAML [6] assuming the relations of ((cow, deer), hippopotamus) as shown in Figure 1. The independent origin hypothesis shown in left side of Figure 1 is compared with the common origin hypothesis shown in the right with the criterion of AIC [3]. Substitution models used in this work are as follows: JC [7], K80 [8], HKY [9], GTR [10, 11], K80+Γ [8, 12], HKY+Γ [9, 12], and GTR+Γ [10–12] for nucleotide substitutions, and Poisson, JTT [13], mtmam [14], Poisson+Γ [12], JTT+F+Γ [12, 13, 15], mtmam+F+Γ [12, 14, 15] models for amino acid substitutions. CLUSTAL W [16] was used for the alignment with various values for gap open penalty (GOP) and gap extension penalty (GEP). The default values of (GOP, GEP) are (15, 6.66) for nucleotide sequences and (10, 0.1) for amino acid sequences, and the default values for amino acid sequences were used in preparing the data sets used in [1], in which only amino acid sequences were analyzed.

## 3. Results and Discussion

The result of the analysis in the nucleotide level is given in Table 1. Without alignment, JC, K80+Γ, HKY+Γ, and GTR+Γ models prefer the common origin hypothesis, while K80, HKY, and GTR models prefer the independent origins hypothesis. The best model with respect to AIC is the GTR+Γ model, and it prefers the common origin. Then, sequences aligned with CLUSTAL W with various GOP and GEP values were analyzed. Larger values of GOP and GEP mean stronger penalty for inserting a gap and gap extension, and accordingly the resulting alignment with larger values is closer to the data set without alignment than that produced with smaller values. By changing the GOP and GEP from large to small values, the common origin hypothesis tends to be preferred over the independent origin hypothesis irrespective of the substitution model. Interestingly, such a situation is realized with (GOP, GEP) = (50, 6.66) before the default values of (15, 6.66).

 A similar analysis in the amino acid level is given in Table 2. In this case, the common origin hypothesis is preferred only by the Poisson and JTT models without alignment, while the best model of mtmam+F+Γ prefers the independent origins. The aligned sequences with the default setting also give different results depending on the assumed substitution model; while simple models such as the Poisson, JTT, and Poisson+Γ prefer the common origin hypothesis, the best available model with respect to AIC, the mtmam+F+Γ model, prefers the independent origins. Probably, the stronger preference of the common ancestor hypothesis with the nucleotide level analysis is, as Theobald pointed out, due to the constraints of the genetic code which induce correlations between the sequences that are not due to common ancestry. Particularly in the mammalian mitochondrial protein-encoding genes on the heavy strand used in our analysis, second codon positions are biased toward T, whereas third codon positions are biased towards A and biased against G [5]. Therefore, the strong preference of the common origin hypothesis by the nucleotide analysis is probably due to the constraints of the genetic code. However, it is worthwhile to be mentioned that, although the best available substitution model of amino acid analysis without alignment and with alignment of the default setting prefers the independent origin hypothesis, the common origin hypothesis is preferred by some substitution models. This raises a serious problem as to the effectiveness of the formal test. Theobald used a similar data set of amino acid sequences as that of Brown et al. [1], who used the CLUSTALW [16] with default settings to align individual protein data sets. Actually, Theobald [2] used another program called ProbCons [17] instead of CLUSTALW in aligning the sequences, but the difference should not be critically important for our arguments.

 Since *cytb* and *nd2* encoded on the heavy strand of mitochondrial DNA have similar amino acid compositions [18], this may induce correlations between these sequences that are not due to common ancestry. This illuminates another flaw in Theobald's analysis; that is, he did not take account of the possibility of convergent evolution as discussed by us [4]. While the examples discussed in [4] were in convergence due to requirement of similar function and to adaptation to similar environment, there is another type of convergence, that is, convergence to similar amino acid composition, which can be achieved by many different ways. A similar amino acid composition between *cytb* and *nd2* may not be bona fide convergence but may only represent constraints due to coexistence of the two genes in the same genome but effectively represents a similar situation of convergent evolution.

 As for the bias caused by the alignment, theoretically it can be solved by including the alignment procedure in the framework of maximum likelihood tree estimation [19–21]. Most current alignment programs treat alignment and phylogeny separately, whereas in fact they are interdependent. When a practical method to estimate both alignment and phylogeny simultaneously in the framework of maximum likelihood is developed, we would be able to compare AIC between the UCA and the independent origin hypotheses by taking account of log-likelihood for insertion/deletion process without any bias for the UCA hypothesis. On the other hand, however, it seems not easy to take account of the possibility of convergent evolution, since any currently used maximum likelihood method assumes a stochastic process representing diversifying evolution, and it is difficult to take account of convergent evolution in this framework. A completely new paradigm might be needed to finally solve the problem which Theobald challenged. Notwithstanding these problems in proving the existence of UCA by statistical testing, it is true that there is strong circumstantial evidence for its existence [2].

Charles Darwin wrote in *On the Origin of Species* [22] as follows: “I should infer from analogy that probably all the organic beings which have ever lived on this earth have descended from someone primordial form, into which life first breathed”. Darwin seems to have discarded multiple origins of life on Earth. However, as Theobald [2] correctly noted, the theory of UCA allows for the possibility of multiple independent origins of life [23, 24]. The UCA hypothesis simply states that all extant life on Earth has descended from a single common ancestral species. There must have been a huge amount of extinctions during the course of the history of life, and there is no way to know what kinds of life became extinct during the early evolution of life. Still, it seems likely that a huge amount of trials and errors of different forms occurred during the emergence of life and that UCA if existed was just one of them. Further, as argued by Raup and Valentine [24], the probability of survival of life is low unless there are multiple origins. Even if the UCA hypothesis holds, the survival of the particular form of life does not imply that it was unique or superior.

## Figures and Tables

**Table:** (a)

Model	No alignment (1038 bp)		(GOP, GEP) = (100, 100) (1026 bp)		(GOP, GEP) = (50, 6.66) (1029 bp)	
	Independent	Common	Independent	Common	Independent	Common
JC	11043.8	11005.5^†^	10876.9	10844.5^†^	10935.0	10862.9^†^
K80	10820.8^†^	10821.2	10669.3	10662.2^†^	10727.6	10684.4^†^
HKY	10398.6^†^	10414.7	10255.3^†^	10266.6	10309.7	10294.4^†^
GTR	10307.5^†^	10320.4	10186.5^†^	10192.1	10242.4	10224.3^†^
K80+Γ	10789.5	10723.4^†^	10637.5	10562.7^†^	10695.7	10650.4^†^
HKY+Γ	10329.8	10274.8^†^	10186.4	10119.4^†^	10239.7	10228.4^†^
GTR+Γ	10271.9	10216.4 ^†^	10129.5	10066.6 ^†^	10184.1	10168.6 ^†^

Homology*	0.314		0.317		0.349	

**Table:** (b)

Model	(GOP, GEP) = (30, 6.66) (1025 bp)		(GOP, GEP) = (15, 6.66) (999 bp)		(GOP, GEP) = (3, 6.66) (974 bp)	
	Independent	Common	Independent	Common	Independent	Common
JC	10890.6	10802.2^†^	**10592.4**	**10409.2** ^†^	10262.1	9865.7^†^
K80	10684.6	10623.3^†^	**10395.0**	**10221.3** ^†^	10056.9	9613.1^†^
HKY	10271.8	10241.0^†^	**9991.1**	**9875.0** ^†^	9645.8	9283.2^†^
GTR	10204.9	10170.3^†^	**9921.1**	**9820.4** ^†^	9585.0	9234.3^†^
K80+Γ	10652.5	10577.5^†^	**10363.0**	**10188.2** ^†^	10028.1	9595.4^†^
HKY+Γ	10202.4	10162.0^†^	**9920.5**	**9817.6** ^†^	9580.9	9249.5^†^
GTR+Γ	10146.3	10099.7 ^†^	**9863.6**	**9768.5** ^†^	9531.1	9201.7 ^†^

Homology*	0.360		**0.419**		0.504	

AICs of each model comparing the independent and common origin hypotheses were shown. In the comparison between the two hypotheses, the hypothesis with lower AIC was indicated by ^†^. The substitution model with the minimal AIC in each data set was indicated by an underline. Default values of GOP and GEP were indicated in bold fonts.

*Homology between *cytb* and *nd2* alignments, which is defined by 1–(average *p*-distance between *cytb* and *nd2*).

**Table 2:** Formal tests of the common ancestry between *cytb* and *nd2* based on the amino acid sequence data sets aligned with various values of gap penalties (GOP and GEP).

Model	No alignment (346 aa)		(GOP, GEP) = (100, 100) (338 aa)		(GOP, GEP) = (15, 6.66) (342 aa)		(GOP, GEP) = (10, 0.1) (330 aa)		(GOP, GEP) = (1, 0.1) (313 aa)	
	Independent	Common	Independent	Common	Independent	Common	Independent	Common	Independent	Common
Poisson	5934.3	5933.5^†^	5748.6	5745.8^†^	5856.9	5838.6^†^	**5664.9 **	**5638.0** ^†^	5403.1	5288.6^†^
Poisson+Γ	5922.0^†^	5933.5	5735.9^†^	5740.6	5843.9	5832.3^†^	**5651.7 **	**5639.0** ^†^	5392.7	5288.5^†^
JTT	5591.5	5586.1^†^	5420.3	5414.0^†^	5515.8	5495.6^†^	**5335.5 **	**5276.4** ^†^	5080.2	4879.8^†^
mtmam	5247.4^†^	5252.5	5083.1^†^	5090.8	5174.7^†^	5176.0	**4995.4 **	**4989.9** ^†^	4754.3	4688.6^†^
JTT+F+Γ	5304.3^†^	5325.8	5133.7^†^	5152.8	5226.8^†^	5231.7	**5044.8 **	**5034.2** ^†^	4809.5	4682.4^†^
mtmam+F+Γ	5248.1 ^†^	5272.3	5082.6 ^†^	5107.7	5174.6 ^†^	5185.4	**4995.0** ^†^	**4995.6 **	4759.7	4678.7 ^†^

Homology*	0.077		0.083		0.107		**0.123 **		0.216	

AICs of each model comparing the independent and common origin hypotheses were shown. In the comparison between the two hypotheses, the hypothesis with lower AIC was indicated by ^†^. The substitution model with the minimal AIC in each data set was indicated by an underline. Default values of GOP and GEP were indicated in bold fonts.

*Homology between *cytb* and *nd2* alignments, which is defined by 1–(average *p*-distance between *cytb* and *nd2*).
